# Cross-sectional and longitudinal neuroanatomical profiles of distinct clinical (adaptive) outcomes in autism

**DOI:** 10.1038/s41380-023-02016-z

**Published:** 2023-03-29

**Authors:** Charlotte M. Pretzsch, Dorothea L. Floris, Tim Schäfer, Anke Bletsch, Caroline Gurr, Michael V. Lombardo, Chris H. Chatham, Julian Tillmann, Tony Charman, Martina Arenella, Emily Jones, Sara Ambrosino, Thomas Bourgeron, Guillaume Dumas, Freddy Cliquet, Claire S. Leblond, Eva Loth, Bethany Oakley, Jan K. Buitelaar, Simon Baron-Cohen, Christian F. Beckmann, Antonio M. Persico, Tobias Banaschewski, Sarah Durston, Christine M. Freitag, EU-AIMS/AIMS-2-TRIALS Consortium, Declan G. M. Murphy, Declan G. M. Murphy, Christine Ecker

**Affiliations:** 1 Department of Forensic and Neurodevelopmental Sciences, Institute of Psychiatry, Psychology and Neuroscience, King’s College London, London, UK; 2 Methods of Plasticity Research, Department of Psychology, University of Zurich, Zurich, Switzerland; 3 Department of Cognitive Neuroscience, Donders Institute for Brain, Cognition and Behaviour, Radboud University Nijmegen Medical Centre, Nijmegen, Netherlands; 4 Department of Child and Adolescent Psychiatry, Psychosomatics and Psychotherapy, University Hospital Frankfurt, Goethe University Frankfurt, Frankfurt am Main, Germany; 5 Laboratory for Autism and Neurodevelopmental Disorders, Center for Neuroscience and Cognitive Systems @UniTn, Istituto Italiano di Tecnologia, Rovereto, Italy; 6 F. Hoffmann La Roche, Innovation Center Basel, Basel, Switzerland; 7 Clinical Child Psychology, Department of Psychology, Institute of Psychiatry, Psychology and Neuroscience, King’s College London, London, UK; 8 Department of Human Genetics, Radboud University Medical Centre, Nijmegen, The Netherlands; 9 Centre for Brain & Cognitive Development, University of London, London, UK; 10 University Medical Center Utrecht, Utrecht University, Utrecht, Netherlands; 11 Human Genetics and Cognitive Functions, Institut Pasteur, UMR3571 CNRS, IUF, Université Paris Cité, Paris, France; 12 CHU Sainte-Justine Research Center, Department of Psychiatry, University of Montreal, Montreal, QC Canada; 13 Autism Research Centre, Department of Psychiatry, University of Cambridge, Cambridge, UK; 14 Child and Adolescent Neuropsychiatry, Department of Biomedical, Metabolic and Neural Sciences, University of Modena and Reggio Emilia, Modena, Italy; 15 Department of Child and Adolescent Psychiatry, Central Institute of Mental Health, Medical Faculty Mannheim, University of Heidelberg, Mannheim, Germany

**Keywords:** Autism spectrum disorders, Neuroscience, Genetics

## Abstract

Individuals with autism spectrum disorder (henceforth referred to as autism) display significant variation in clinical outcome. For instance, across age, some individuals’ adaptive skills naturally improve or remain stable, while others’ decrease. To pave the way for ‘precision-medicine’ approaches, it is crucial to identify the cross-sectional and, given the developmental nature of autism, longitudinal neurobiological (including neuroanatomical and linked genetic) correlates of this variation. We conducted a longitudinal follow-up study of 333 individuals (161 autistic and 172 neurotypical individuals, aged 6–30 years), with two assessment time points separated by ~12–24 months. We collected behavioural (Vineland Adaptive Behaviour Scale-II, VABS-II) and neuroanatomical (structural magnetic resonance imaging) data. Autistic participants were grouped into clinically meaningful “Increasers”, “No-changers”, and “Decreasers” in adaptive behaviour (based on VABS-II scores). We compared each clinical subgroup’s neuroanatomy (surface area and cortical thickness at T1, ∆T (intra-individual change) and T2) to that of the neurotypicals. Next, we explored the neuroanatomical differences’ potential genomic associates using the Allen Human Brain Atlas. Clinical subgroups had distinct neuroanatomical profiles in surface area and cortical thickness at baseline, neuroanatomical development, and follow-up. These profiles were enriched for genes previously associated with autism and for genes previously linked to neurobiological pathways implicated in autism (e.g. excitation-inhibition systems). Our findings suggest that distinct clinical outcomes (i.e. intra-individual change in clinical profiles) linked to autism core symptoms are associated with atypical cross-sectional and longitudinal, i.e. developmental, neurobiological profiles. If validated, our findings may advance the development of interventions, e.g. targeting mechanisms linked to relatively poorer outcomes.

## Introduction

Autism spectrum disorder (henceforth referred to as autism), estimated to occur in ~1 out of 54 individuals [1], is one of the most common neurodevelopmental conditions. Autism is characterised by social-communication difficulties and restricted and repetitive patterns of interests and behaviours [2]. These symptoms can converge to disrupt adaptive behaviour, i.e. “the development and application of the abilities required for the attainment of personal independence and social sufficiency” [3]. Accordingly, difficulties in adaptive behaviour are thought to represent a distinctive feature of autism, compared to other neurodevelopmental conditions [4]; play a crucial role in autism diagnosis (e.g. measures of adaptive behaviour improve diagnostic accuracy beyond that provided by gold-standard instruments [5]) and intervention planning [4, 6]; have been recommended as an outcome measure by both the Food and Drug Administration [FDA] and stakeholders) in both children and adults [7, 8]; and so have been used as the primary target in numerous clinical trials across the age-span.

Combined, autism core and associated difficulties (including disrupted adaptive behaviour) can significantly affect individuals and society. For instance, only 12% of autistic adults are in full-time paid work [9]. Also, a recent study estimated the cost of supporting autistic individuals with (or without) intellectual disability over their lifespan at $2.4 million ($1.4 million) in the United States and £1.5 million (£0.92 million) in the United Kingdom [10]. Hence there is an urgent need for effective interventions and support strategies in autism.

However, clinical trials addressing core symptoms in autism have largely failed [11]. A key reason for this is the substantial clinical and biological heterogeneity within autism. For instance, across the lifespan, some individuals’ adaptive behaviour skills naturally improve or remain stable, while others’ decrease [12]. This natural variation in clinical outcome (i.e. intra-individual change in clinical profiles over time) may distort the results of clinical trials. Also, it highlights the need to develop ‘precision medicine’ approaches by gaining a better understanding of the mechanisms that contribute to differences in adaptive clinical outcomes. In the future, this knowledge may help to e.g. tailor treatments more effectively to those individuals with a relatively poor prognosis.

Previous research investigated how (change in) adaptive behaviour is linked to variation in cognitive ability, brain functional connectivity and neuroanatomy. For example, studies reported that relatively poor adaptive behaviour and outcome may be underpinned by reduced overall cognitive ability (i.e. the intelligence quotient (IQ) [13, 14]); and/or particular resting state functional connectivity patterns [15]. Also, we recently demonstrated that autistic subgroups with distinct future adaptive outcomes differed in baseline neuroanatomy (including cortical thickness, surface area, and cortical volume) in multiple brain regions relevant to autism and enriched for genes relevant to autism [16]. Moreover, in these regions, greater deviation from the neurotypical neuroanatomical profile predicted poorer adaptive outcome at the individual level. Together, these studies represent important first steps, but they had several limitations. For instance, the relationship between IQ and adaptive outcome may be complex and vary across individuals, e.g. based on sex, age, or cognitive ability [17, 18]. Hence, some individuals with high IQ also have poor adaptive outcomes [19]. Also, resting state functional connectivity patterns were not always specific to individuals with particular adaptive outcomes (maximum specificity 67%; [15]). Further, in our previous work [16], we only examined neuroanatomy cross-sectionally (at baseline); and compared neuroanatomy between different autistic subgroups. However, autism is a developmental condition where not only clinical, but also associated neuroanatomical, development may vary—both within autism and in autism compared to neurotypical individuals (e.g. reviewed in refs. [20, 21]).

Hence, if we want to better understand the neuroanatomical correlates of variation in adaptive outcome, we need to examine them not only cross-sectionally, but also longitudinally (i.e. across time and age); and in autistic subgroups compared to non-autistic individuals (henceforth referred to as neurotypicals).

Therefore, here we extend our previous work [16] by investigating if differences in adaptive outcome in autism are paralleled by differences (compared to neurotypicals) in neuroanatomical developmental trajectories. We leveraged one of the largest deep-phenotyped longitudinal autism datasets worldwide (EU-AIMS Longitudinal European Autism Project [22]) and our final sample included 333 individuals (161 autistic and 172 neurotypical individuals, age 6–30 years). We collected longitudinal adaptive behavioural (Vineland Behaviour Scale-II, VABS-II) and neuroanatomical (structural magnetic resonance imaging) data at two assessment time points (T1 and T2) separated by ~12–24 months. Following recently published criteria [23], we grouped ASD individuals into three clinically meaningful outcome groups—“Increasers”, “No-changers”, and “Decreasers” in adaptive behaviour (based on VABS-II scores, as in [16]). Note that we chose to group individuals based on the VABS-II, because, for the VABS-II (unlike for other metrics, such as the gold-standard Autism Diagnostic Observation Schedule [ADOS] and the Autism Diagnostic Interview-Revised [ADI-R]), there exists an empirical measure of the Minimal Clinically Important Difference (MCID). This MCID quantifies the amount of change required to be clinically (rather than statistically) meaningful; is approved by the FDA [7]; and has previously been used to quantify clinical outcome in autism [16]. First, to identify the clinical outcome groups’ cross-sectional and longitudinal neuroanatomical profiles, we compared each group’s neuroanatomy (surface area and cortical thickness at T1, ∆T (intra-individual neuroanatomical change), and T2) to that of the neurotypicals. Next, we explored the neuroanatomical profiles’ potential genomic (genetic and transcriptomic) associates. Specifically, we leveraged the Allen Human Brain Atlas [24] to identify genes whose spatial expression maps resembled our patterns of neuroanatomical differences between autistic subgroups and neurotypicals. We then examined the enrichment of those genes for genes broadly associated with autism; and for genes linked to various biological pathways implicated in the aetiology of autism. We hypothesised that, compared to the neurotypicals, each outcome group would present with distinct cross-sectional and longitudinal neuroanatomical profiles. We further expected that these neuroanatomical profiles would be enriched for genes previously found to be associated with variable (adaptive behaviour-related) neuroanatomy in autism.

## Materials and methods

### Study design

Our data was part of the Longitudinal European Autism Project (LEAP) described in [22]. We included participants if they or their parents/guardians were able to provide informed written or verbal consent/assent to their participation in this study. Our study was approved by national and local ethics review boards at all study sites and carried out to Good Clinical Practice (ICH GCP) standards. See the Supplementary Material for a full description of clinical assessments, inclusion and exclusion criteria, and ethics review boards.

### Measures of adaptive functioning using the VABS-II

The autistic participants’ adaptive behaviour was assessed by trained and reliable interviewers using the VABS-II [25], which assesses a person’s current level of everyday functioning across three domains (communication, daily living skills, and socialisation). We calculated age-normed standard scores (mean = 100, standard deviation = 15) for each domain and generated composite scores (i.e. total degree of impairment across all three domains) at T1 and T2. We then quantified the change between T1 and T2 (∆ = T2–T1) and used recently published estimates of what constitutes an MCID [23], to group autistic individuals into three adaptive clinical outcome groups: those whose scores could be said to meaningfully improve (“Increasers”; ∆V ≥ 4), showed no meaningful change/stasis (“No-changers”; −4 < ∆V < 4), and those whose scores declined (“Decreasers”; −4 ≥ ∆V). Note that the MCID quantifies the amount of change required to be clinically, rather than statistically, meaningful. Accordingly, the MCID has been supported as a means to evaluate (treatment) outcomes, including by the FDA [7]. Note that VABS-II scores are age-normed and should therefore be interpreted considering the expected (‘normative’) value at a given age. For instance, an individual’s adaptive behaviour skills may increase between age at T1 and age at T2; however, if such an increase is to be expected during this period, the individual will be classified as a “No-changer” (i.e. not changing in relation to the age-normed value), and their (age-normed) VABS-II scores at T1 and T2 may be the same. For more detail, refer to the Supplementary Material.

### MRI data acquisition

We used standard 3 T magnetic resonance imaging (MRI) scanners to obtain high-resolution T1-weighted volumetric structural images with full head coverage (field of view = 27 cm, slice thickness = 1.2 mm, in-plane resolution = 1.1*1.1 mm^2^, for more detail see ref. [16]).

### Cortical reconstruction using FreeSurfer

Images were (pre)processed using well-validated, automated procedures (see Supplementary Material). Of the initial 709 scans at baseline, we retained 639 scans. Of the initial 459 scans at follow-up, we retained 428 images. After excluding all participants who did not have both T1 and T2 structural data, and those autistic individuals who did not have both T1 and T2 adaptive behavioural data, our final sample consisted of 333 individuals (161 autistic and 172 neurotypical individuals) (Table 1). We computed vertex-wise (site-corrected) cross-sectional and longitudinal measures of surface area and cortical thickness (for more information, see Supplementary Material).

**Table 1:** Demographics (at T1, unless otherwise specified) and total brain measures.

Measure	Decreasers *n* = 53	No-changers *n* = 42	Increasers *n* = 66	Test Statistic (autistic subgroups)		Autism *N* = 161	Neurotypicals *N* = 172	Test statistic (autisticSD vs. neurotypical participants)	
ADI social	16.21 ± 7.3	17.93 ± 5.7	16.29 ± 6.9 (65)	F2,157 = 0.962	*p* = 0.384	1.69 ± 6.7 (160)			
ADI comm	13.26 ± 5.8	14.64 ± 5.7	12.89 ± 5.6 (65)	F2,157 = 1.258	*p* = 0.287	13.48 ± 5.7 (160)			
ADI RRB	3.98 ± 2.8	5.17 ± 2.6	3.52 ± 2.2 (65)	F2,157 = 5.459	*p* = 0.005	4.11 ± 2.6 (160)			
Age (Years)	17.07 ± 6.7	14.68 ± 4.3	18.10 ± 4.7	F2,158 = 5.337	*p* = 0.006	16.87 ± 5.5	16.35 ± 5.7	F1,331 = 0.727	*p* = 0.394
CSS total	5.35 ± 2.9 (52)	5.60 ± 2.8 (40)	4.83 ± 2.5 (63)	F2,152 = 1.090	*p* = 0.339	5.20 ± 2.74 (155)			
CSS SA	6.02 ± 2.8 (52)	6.25 ± 2.6 (40)	5.48 ± 2.5 (63)	F2,152 = 1.187	*p* = 0.308	5.86 ± 2.7 (155)			
CSS RRB	4.77 ± 2.8 (52)	4.63 ± 2.7 (40)	4.29 ± 2.9 (63)	F2,152 = 0.450	*p* = 0.638	4.54 ± 2.8 (155)			
FSIQ	95.75 ± 18.9	105.06 ± 22.6	104.63 ± 17.8	F2,158 = 3.832	*p* = 0.024	101.82 ± 19.8	107.05 ± 16.5	F1,331 = 6.888	*p* = 0.009
ID	9	5	5	χ22 = 2.499	*p* = 0.287	19	11	χ21 = 2.965	*p* = 0.085
Mean CT (mm)	2.68 ± 0.1	2.71 ± 0.1	2.67 ± 0.1	F2,158 = 1.586	*p* = 0.208	2.69 ± 0.1	2.69 ± 0.1	F1,331 = 0.012	*p* = 0.912
Sex	25 F, 28 M	6 F, 36 M	19 F, 47 M	χ22 = 12.103	*p* = 0.002	50 F, 111 M	64 F, 108 M	χ21 = 1.399	*p* = 0.250
Time (yrs)	1.60 ± 0.3	1.60 ± 0.3	1.64 ± 0.2	F2,158 = 0.494	*p* = 0.611	1.62 ± 0.3	1.59 ± 0.3	F1,331 = 1.041	*p* = 0.308
Total SA (cm2)	2230.11 ± 271.08	2349.98 ± 159.96	2308.22 ± 228.0	F2,158 = 3.459	*p* = 0.034	2293.40 ± 232.0	2316.47 ± 225.0	F1,331 = 0.848	*p* = 0.358
T1 V Comm	81.60 ± 18.3	77.00 ± 12.5	73.74 ± 13.5	F2,158 = 4.031	*p* = 0.020	77.18 ± 15.3			
T1 V Daily living	77.98 ± 18.7	76.90 ± 15.4	71.86 ± 12.4	F2,158 = 2.642	*p* = 0.074	75.19 ± 15.6			
T1 V Social	73.38 ± 14.9	71.98 ± 11.2	70.55 ± 15.4	F2,158 = 0.582	*p* = 0.560	71.85 ± 14.2			
T1 V Standard	75.60 ± 15.2	73.31 ± 10.1	69.50 ± 11.0	F2,158 = 3.717	*p* = 0.026	72.50 ± 12.5			
∆ V Comm	−15.06 ± 13.1	−2.55 ± 6.8	9.15 ± 13.0	F2,158 = 62.752	*p* < 0.001	−1.87 ± 15.6			
∆ V Daily living	−10.40 ± 8.5	0.14 ± 7.4	8.59 ± 8.7	F2,158 = 76.666	*p* < 0.001	0.14 ± 11.6			
∆ V Social	−7.83 ± 9.9	2.45 ± 7.8	12.36 ± 10.1	F2,158 = 66.828	*p* < 0.001	3.13 ± 12.8			
∆ V standard	−11.23 ± 8.0	0.05 ± 2.0	9.86 ± 5.5	F2,158 = 187.437	*p* < 0.001	0.36 ± 10.8			
T2 V Comm	66.55 ± 22.1	74.45 ± 11.3	82.89 ± 15.1	F2,158 = 13.710	*p* < 0.001	75.31 ± 18.3			
T2 V Daily living	67.58 ± 16.9	77.05 ± 16.8	80.45 ± 12.9	F2,158 = 10.668	*p* < 0.001	75.33 ± 16.3			
T2 V Social	65.55 ± 19.9	74.43 ± 11.0	82.91 ± 13.7	F2,158 = 18.497	*p* < 0.001	74.98 ± 17.1			
T2 V Standard	64.38 ± 18.7	73.36 ± 10.8	79.36 ± 11.0	F2,158 = 16.961	*p* < 0.001	72.86 ± 15.3			

Data are expressed as mean ± standard deviation (*n*, unless as specified at the top of the column).

*ADI* autism diagnostic interview (comm: communication subscale, *rrb* restricted and repetitive behaviour subscale, *social* social subscale), *CSS* autism diagnostic observation schedule calibrated severity score (*sa* social affect subscale, *rrb* restricted and repetitive behaviour subscale, *total* overall score), *CT* cortical thickness, *F* female, *FSIQ* full-scale IQ, *ID* intellectual disability, *M* male, *SA* surface area, *T1* measure at timepoint 1, *T2* measure at timepoint 2, *V* Vineland Adaptive Behaviour Scale (*comm* communication domain, *daily living* daily living domain, *social* social domain, *standard* composite score), *∆* measurement of change between timepoint 1 and 2.

*P* values are not corrected for multiple comparisons.

### Statistical analyses

First, we examined differences in neuroanatomy at T1 (baseline) between the neurotypicals and each outcome group. We included group and sex as factors; and linear (surface area/cortical thickness) and quadratic (cortical thickness) age at T1 (as in e.g. [16],), IQ, and total brain measures (total surface area, mean cortical thickness) as continuous covariates. Second, we examined differences in intra-individual change in neuroanatomy between T1 and T2 between the neurotypicals and each outcome group. We used separate models for each cortical feature that included the terms above and also corrected for the interaction between age at T1 and the follow-up duration (∆T). Third, we investigated differences in neuroanatomy at T2 (follow-up) between the neurotypicals and each outcome group. We performed separate models as specified above, while correcting for age at T2. We corrected for multiple comparisons across the whole brain using random-field theory (RFT)-based cluster-correction for non-isotropic images (cluster-forming and cluster-*p* value threshold both <0.01, two-tailed) [26]. As surface area and cortical thickness are thought to have distinct neurobiological underpinning mechanisms (e.g. [27]), we treated them as separate analyses and did not correct for multiple comparisons across these two features. Also, we did not correct for multiple comparisons across the three subgroups, as we treated them as clinically separate (for more information, see Supplementary Material and ref. [16, 28]). To establish the robustness of our results in view of additional potential confounders, we repeated our analyses (i) while correcting for medication; (ii) while not controlling for total brain measures; and (iii) while excluding individuals with intellectual disability. To explore the generalisability of our results to other cognitive-behavioural features associated with adaptive behaviour, we repeated our analyses using different approaches to stratify autistic individuals into clinical outcome subgroups. In particular, we grouped individuals into “Increasers”, “No-changers” and “Decreasers” based on change in (i) each of the VABS-II domains, i.e. communication, daily living, and social skills; (ii) the ADOS social domain; and (iii) the ADOS restricted and repetitive behaviour domain. We acknowledge that analyzing change in these measures in conjunction with a cut-off is not a widely used approach to assess clinical development longitudinally. Therefore, we highlight that these analytical steps were taken only as a secondary and exploratory means to investigate the relationship between our primary results (computed using the VABS-II) and those results obtained using alternative (and autism core symptom-related) measures. To evaluate the association between adaptive outcome and neuroanatomy using a dimensional (rather than categorical) approach, we assessed the effect of change in adaptive behaviour on neuroanatomy across autistic subgroups. Finally, to further explore the impact of age, we repeated our analyses while stratifying our sample into age groups (children, adolescents, and adults). (For more information, see Supplementary Material).

Next, we aimed to link our neuroanatomical results to putative genomic (genetic and transcriptomic) mechanisms. First, we identified genes expressed in spatial patterns similar to the neuroanatomical differences between autistic subgroups and neurotypicals using the Allen Human Brain Atlas (AHBA) [24]. Second, we tested the enrichment of these identified genes. We restricted our enrichment analyses a priori to a set of genes that were selected because of their previous implication in autism and adaptive behaviour. We opted for this hypothesis-driven approach because it allowed us to investigate a broad set of genes (genetically and transcriptomically) linked to autism etiology, and because it increased our statistical power. However, the trade-off of our approach was that we were limited in discovering enrichment beyond our chosen gene sets; and we encourage future work that extends our analyses to additional gene sets. In particular, we evaluated how the identified genes overlapped with genes that have previously been associated with autism at the genetic and transcriptomic level [29–32] and that we have previously linked to cross-sectional neuroanatomical variation in autism [16]. We corrected our analyses for multiple comparisons across all subgroup contrasts and gene sets (*p*_FDR_ < 0.05). For more detailed information, see [16, 33] and the Supplementary Material. To examine the robustness of our findings, we repeated our analyses using a more restrictive background list of genes specifically estimated to be expressed in cortical tissue [34]. Also, we extended our analyses to test the association between the observed neuroanatomical differences and specific (developmentally relevant) cell types and neurobiological processes linked to both autism and adaptive behaviour. Specifically, we examined enrichment for three gene sets of interest: (i) genes expressed prenatally in specific cell types; (ii) genes linked to excitatory-inhibitory pathways; and (iii) microglial immune genes.

## Results

### Demographics

Note that, to increase the generalisability of our results, we aimed to recruit a broad and representative number of participants. For instance, in both groups we included individuals with and without intellectual disability and participants across age (i.e. from childhood to adulthood), Also, the autism group comprised individuals with a wide range of symptom severity. Autistic subgroups and neurotypicals did not differ significantly in age, sex, total surface area, mean cortical thickness, and the time between visits. However, as expected, FSIQ was significantly higher in neurotypicals Table 1.

Within autism, subgroups did not differ significantly in Autism Diagnostic Interview-Revised (ADI-R) [35] social and communication measures, Autism Diagnostic Observation Schedule 2 (ADOS-2) [36] Calibrated Severity Scores (CSS), T1 VABS (daily living and social domain) scores, mean cortical thickness, and time between visits. Nonetheless, in addition to VABS change scores (which is how autistic subgroups were derived), groups differed in ADI restricted and repetitive behaviour scores (Increasers < Decreasers < No-changers), FSIQ (Decreasers < Increasers < No-changers), sex, T1 VABS (communication domain and total) scores (Increasers < No-changers < Decreasers), T2 VABS scores (Decreasers < No-changers < Increasers), and total surface area (Decreasers < Increasers < No-changers) (see Table 1; information on medication: Table S4).

### Neuroanatomical differences

#### Primary analyses

**Fig. 1:**
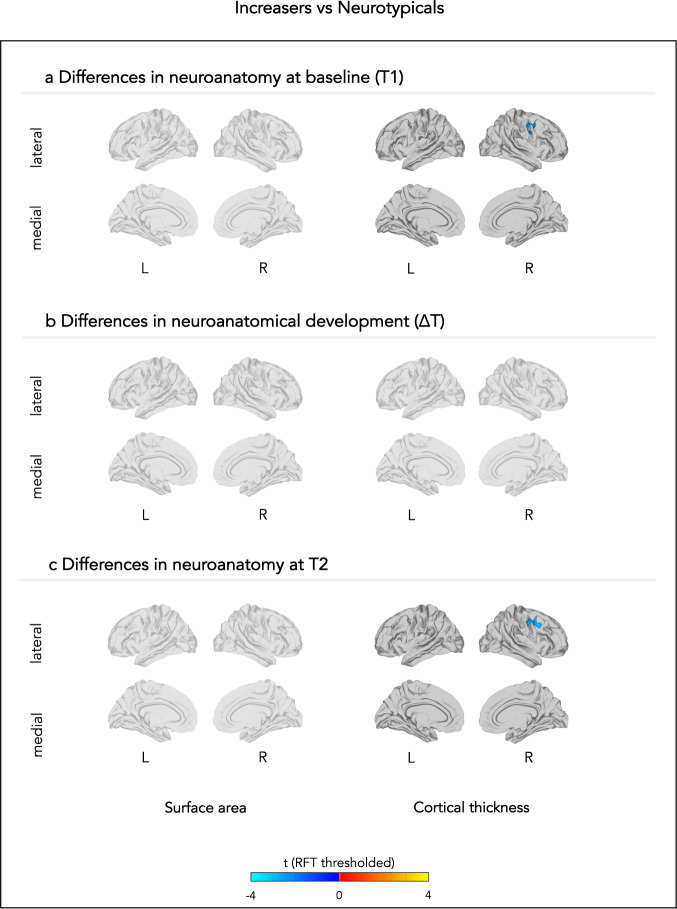
Neuroanatomical differences between neurotypicals and those individuals whose adaptive behavioural scores increased. The figure displays between-group differences in (**a**) neuroanatomy at baseline (T1), (**b**) neuroanatomical development between T1 and T2, and (**c**) neuroanatomy at follow-up (T2). Each row displays random-field theory (RFT)-corrected t-values. L left, R right.

**Fig. 2:**
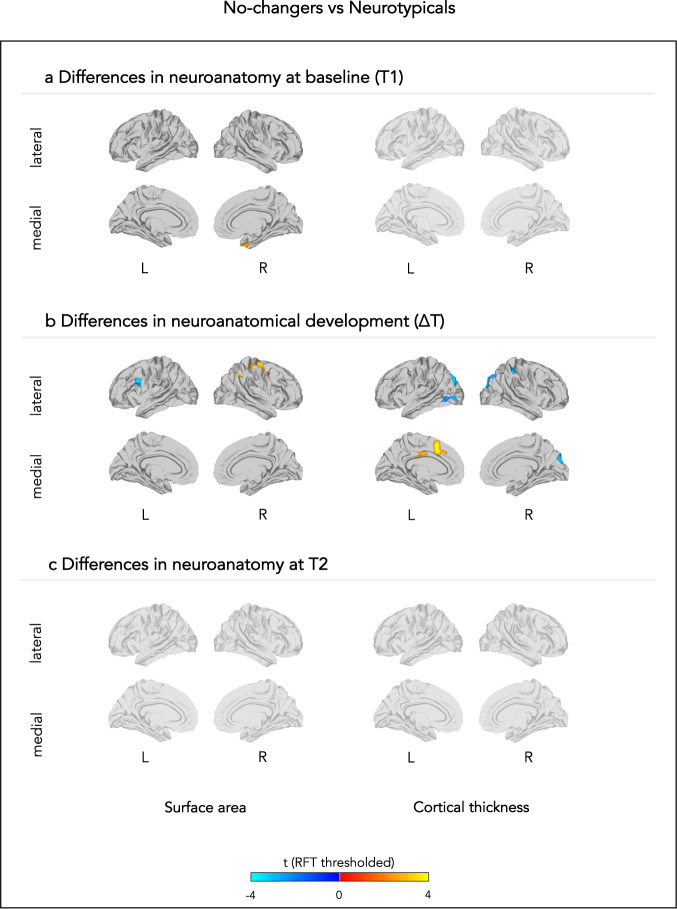
Neuroanatomical differences between neurotypicals and those individuals whose adaptive behavioural scores did not change. The figure displays between-group differences in (**a**) neuroanatomy at baseline (T1), (**b**) neuroanatomical development between T1 and T2, and (**c**) neuroanatomy at follow-up (T2). Each row displays random-field theory (RFT)-corrected t-values. L left, R right.

**Fig. 3:**
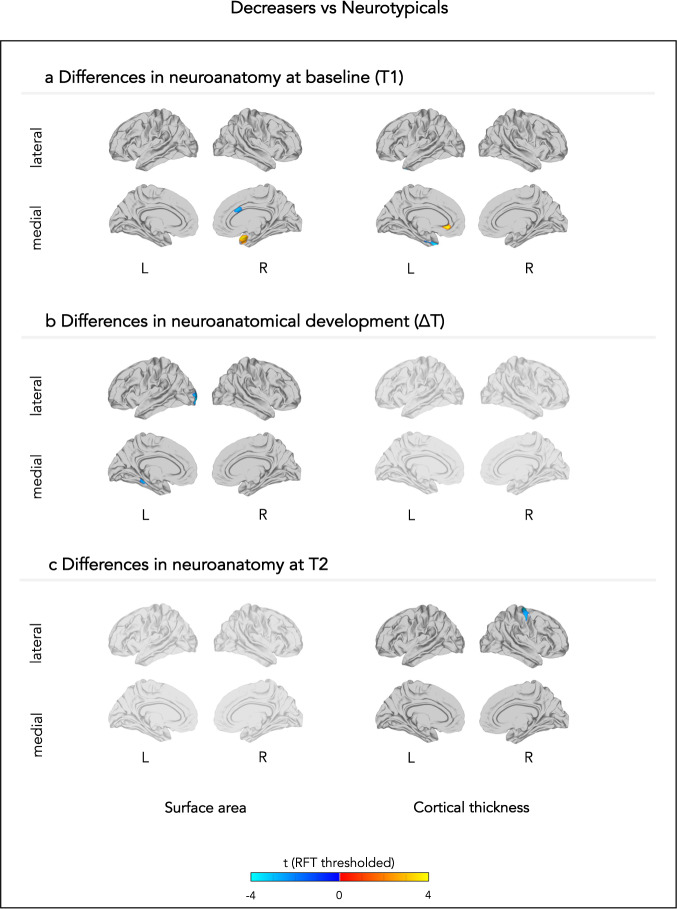
Neuroanatomical differences between neurotypicals and those individuals whose adaptive behavioural scores decreased. The figure displays between-group differences in (**a**) neuroanatomy at baseline (T1), (**b**) neuroanatomical development between T1 and T2, and (**c**) neuroanatomy at follow-up (T2). Each row displays random-field theory (RFT)-corrected t-values. L left, R right.

Briefly, autistic subgroups and neurotypicals displayed neuroanatomical differences at T1, ∆T, and T2 in frontal, temporal, parietal, and occipital regions that are associated with adaptive behaviour and implicated in autism. Increasers (compared to neurotypicals) had largely ‘typical’ neuroanatomical profiles. Specifically, the group showed no differences in cross-sectional and longitudinal surface area, or in longitudinal cortical thickness. However, the group had lower frontal cortical thickness at both T1 and T2 (Fig. 1). No-changers (compared to neurotypicals) showed both cross-sectional and longitudinal atypicality. Specifically, the group had greater temporal surface area at T1; both greater and lower ∆surface area in distinct frontal regions; and greater ∆surface area in parietal regions. At T2, No-changers no longer differed in surface area. No-changers displayed no differences in cortical thickness at T1 or T2; but greater ∆cortical thickness in frontal and posterior cingulate regions, and lower ∆cortical thickness in parietal and occipital regions (Fig. 2). Decreasers (compared to neurotypicals) also showed both cross-sectional and longitudinal differences. In particular, Decreasers had greater temporal and lower anterior cingulate surface area at T1; reduced parietal, occipital, and temporal ∆surface area; but no differences in surface area at T2. Further, the group showed greater frontal cortical thickness and lower temporal cortical thickness at T1; no differences in ∆cortical thickness; and reduced frontal cortical thickness at T2 (Fig. 3). Results are also summarised in more detail in the Supplementary Material in Tables S1–3 (uncorrected T-values: Figs. S1–3; effect sizes: Figs. S4–6).

#### Secondary analyses

Secondary analyses established that our results remained robust in view of additional potential confounders, including correcting for medication effects (Figs. S7–9); not covarying for total brain measures (Figs. S7–9); and when excluding individuals with intellectual disability (Figs. S10–12). This suggests that our results were not confounded by these measures. Further, our secondary analyses demonstrated that neuroanatomical differences between neurotypicals and autistic subgroups were also present when employing alternative strategies to identify clinical subgroups. Specifically, we obtained results similar to our main findings when comparing neuroanatomy between neurotypicals and clinical subgroups (“Increasers”, “No-changers”, and “Decreasers”) based on change in (i) each of the VABS-II domains, (ii) the ADOS social domain, and (iii) the ADOS restricted and repetitive behaviour domain (Figs. S13–21). Also, we identified neuroanatomical regions associated with adaptive outcome across autistic subgroups (Fig. S22); as well as neuroanatomical between-group differences within age groups, i.e. children, adolescents, and adults (Figs. S23–28).

### Genomic associates

#### Primary analyses

**Fig. 4:**
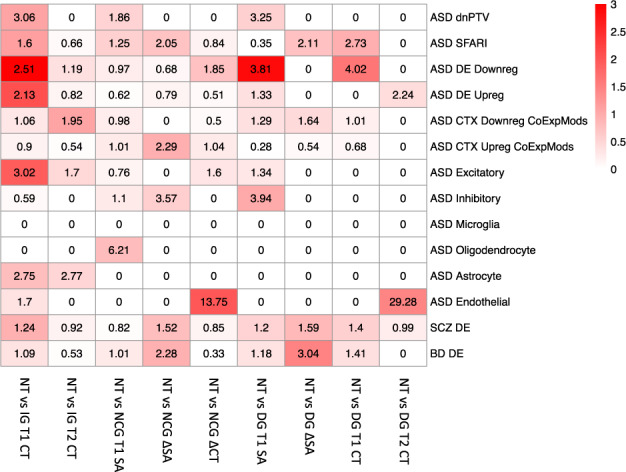
Genetic correlates of neuroanatomical variability: enrichment analyses for cortical phenotypes (y-axis, rows) by autism-associated gene lists (x-axis, columns). Tile colours indicate FDR q-values. Tile labels indicate enrichment odds ratios. CT cortical thickness, ∆ change between T1 and T2, DG Decreasers, IG Increasers, NCG No-changers, NT Neurotypicals, SA surface area, T1 timepoint 1, T2 timepoint 2.

Neuroanatomical differences between autistic subgroups and neurotypicals were associated with genomic mechanisms implicated in autism and previously linked to cross-sectional neuroanatomical variation within autism [16]. Specifically, differences between Increasers and neurotypicals in cortical thickness at T1, and differences between Decreasers and neurotypicals in surface area at T1 corresponded to spatial expression patterns of gene sets previously reported to be downregulated in autism (cortical thickness: OR = 2.51, *p*_FDR_ = 0.006; surface area: OR = 3.81, *p*_FDR_ = 0.018) [30]. All other imaging contrasts showed no significant enrichments Fig. 4.

#### Secondary analyses

Our results remained largely unchanged when we repeated our analyses using a more restrictive background of those genes specifically estimated to be expressed in cortical tissue [34] (Fig. S29). Also, secondary analyses demonstrated that our neuroanatomical results were associated with a range of genes linked to specific (developmentally relevant) cell types and neurobiological processes implicated in both autism and adaptive behaviour. First, differences between Increasers and neurotypicals in cortical thickness at T1 were enriched for gene expression associated prenatally with excitatory deep layer II cells (OR = 2.37, *p*_FDR_ = 0.020) and maturing excitatory cells enriched in upper layers (OR = 4.01, *p*_FDR_ = 0.012) [37]. Also, neuroanatomical differences between No-changers and neurotypicals in ∆cortical thickness corresponded with spatial expression patterns of genes linked prenatally to migrating excitatory cells (OR = 15.82, *p*_FDR_ = 0.019) [37] (Fig. S30). Second, neuroanatomical differences between Increasers and neurotypicals in cortical thickness at T2 were associated with spatial expression patterns of genes implicated in GABAergic pathways (OR = 8.73, *p*_FDR_ < 0.001) (Fig. S31). Third, neuroanatomical differences between No-changers and neurotypicals in ∆surface area corresponded with expression patterns of microglial immune genes (OR = 6.63, *p*_FDR_ = 0.013) [38] (Fig. S32). We observed no significant enrichments for other gene sets or between-group contrasts.

## Discussion

Here, we examined the cross-sectional and longitudinal neuroanatomical correlates of adaptive outcome (i.e. intra-individual change in adaptive behaviour across time) over a period of ~1–2 years in autism, as well as their putative associated genomic mechanisms. This study extends our previous research into the cross-sectional neuroanatomical associates of variation in adaptive outcome within autism [16]. Specifically, it demonstrates that autistic subgroups with different adaptive outcomes have distinct neuroanatomical difference profiles (compared to neurotypicals) concerning measures of surface area and cortical thickness (i) at baseline, (ii) in their neuroanatomical development, and (iii) at follow-up. These neuroanatomical profiles were enriched for genes previously reported to be associated with autism itself and for genes linked to specific neurobiological pathways implicated in autism (e.g. excitation-inhibition systems). Taken together, our findings suggest that distinct clinical outcomes related to autism core symptoms are associated with atypical cross-sectional *and* longitudinal (i.e. developmental) neurobiological profiles.

As noted earlier, previous studies in autism have linked adaptive outcome to brain function and structure. For example, we recently reported that adaptive outcome was associated with, and predicted by, neuroanatomical variation within autism (at both the group- and individual level) [16]. However, this previous work was limited to examining cross-sectional predictors of adaptive outcome; whereas autism is a neurodevelopmental condition associated with different (compared to neurotypicals) clinical *and* neuroanatomical development (e.g. see refs. [20, 28, 39, 40]). Therefore, to better understand the neurobiological correlates of adaptive behaviour and outcome, here we examined them both cross-sectionally and longitudinally, i.e. across time and age, and in relation to neurotypicals. Our results suggest that a change in adaptive behaviour is paralleled by not only cross-sectional but also longitudinal neuroanatomical variation. Specifically, autistic subgroups (compared to neurotypicals) displayed distinct neuroanatomical profiles at T1, ∆T, and T2; and these profiles were robust when considering several potential confounders, including age, total brain measures, medication, and intellectual disability (information concerning other types of interventions, education, employment, and living arrangements was not available; and future studies are required to examine how these factors relate to our results).

The observed neuroanatomical profiles were characterised to varying degrees by atypicality in *both* surface area and cortical thickness. However, the atypicality patterns of these features displayed little or no spatial overlap. This is in line with previous evidence that surface area and cortical thickness represent distinct aspects of cortical architecture—with separate developmental origins and roles in brain development [41]. Combined, this suggests that different neurodevelopmental mechanisms underpin variation in discrete aspects of cortical anatomy and that to better understand outcome-related neuroanatomy in autism, it is essential to examine multiple different cortical features across time.

Further, the neuroanatomical differences we observed between autistic subgroups and neurotypicals occurred in regions that have previously been implicated both in autism and in adaptive behaviour. For example, we identified neuroanatomical differences in frontal lobe regions, such as the superior/middle/inferior frontal gyrus, precentral gyrus, premotor cortex and supplementary motor area, and caudal/dorsal anterior cingulate cortex. These regions have previously been noted to be involved in autism and linked to (interpersonal) emotion regulation, facial emotion recognition, and adaptive behaviour in autism and neurotypicals [42–51]. We also identified temporal lobe regions, including the superior temporal gyrus, temporal pole, and parahippocampal gyrus. These regions have been reported to be neuroanatomically different in autism and have been associated with social-emotional cognition (e.g. language and empathy processing) and behavioural adaptation in both autistic and neurotypical populations [42, 46, 52–54]. Parietal regions highlighted in our study included the superior/inferior parietal cortex, postcentral gyrus, and posterior cingulate cortex, which are also frequently reported structures in previous neuroimaging studies: among other functions, they have been linked to social cognition, emotional representation, behavioural evaluation, and decision making in both autistic and neurotypical individuals [44, 55–58]. Occipital regions included the cuneus and lateral occipital cortex. Both have been neuroanatomically implicated in autism, and linked to the processing of empathy, social inclusion/exclusion, and sensitivity to social and emotional cues in autistic and neurotypical individuals [42, 46, 59–61]. Several regions were implicated in more than one between-group contrast. For instance, both No-changers and Decreasers displayed differences in parietal and occipital cortex. Nonetheless, groups differed in how these regions were implicated (i.e. at which timepoint or in which feature). Hence, despite the regional overlap, groups displayed largely distinct neuroanatomical profiles. Taken together, these studies add biological plausibility to our findings by linking the regions where we observed outcome-relevant neuroanatomical variation to adaptive (and related) behaviour and to autism. Specifically, they reinforce the notion that these regions are both structurally and functionally implicated in (the development of) adaptive behaviour in autism. (Note that, as the regions we identified were relatively large and associated with a broad set of functions, it is inherently difficult to relate them to the specific neural mechanisms underlying adaptive behaviour. We further address this difficulty below, when discussing the i) genomic correlates of our results, and the ii) specificity of our neurobiological findings to adaptive behaviour).

Additional research is required to discern if the observed reductions and enlargements in specific neuroanatomical features are primary or secondary, and detrimental or beneficial to (better) adaptive outcome. This is because the mechanistic relationship between neuroanatomical and clinical outcome remains unclear. Previous studies suggest that neuroanatomy may influence adaptive outcome, e.g. by limiting or enhancing the neural substrate available to adaptive behaviour. However, adaptive behaviour may also affect neuroanatomy, e.g. through activity-dependent alterations of synaptic and dendritic spine density [62]. We previously reported that neuroanatomical differences at baseline (i.e. prior to subsequent clinical change) were predictive of adaptive outcome [16]—suggesting that (atypical) neuroanatomical variation may give rise to (atypical) behavioural development. However, these neuroanatomical differences may themselves have been influenced by/resulted from clinical change prior to our study etc. Moreover, clinical and neuroanatomical atypicalities may accumulate and compound each other across the lifespan. Taken together, this suggests that associations between neuroanatomical and clinical outcome need to be understood in the context of life-long developmental trajectories.

The neuroanatomical differences we observed in the autistic subgroups are likely modulated by a variety of genetic and other (e.g. environmental) factors. For instance, previous studies have associated variability in cortical thickness in autism with variation in genes involved in synaptic transmission pathways [63]. Also, we have previously linked adaptive outcome-related cross-sectional neuroanatomical variation between autistic subgroups to gene sets broadly associated with autism [16]. These sets comprised genes involved in key neurobiological pathways in autism, such as neurogenesis, cell proliferation, neuronal development, and synaptic processes [30]. Here, we report that spatial patterns of cross-sectional differences between Increasers/Decreasers and neurotypicals were associated with these same gene sets. This suggests that (atypical) clinically meaningful change in behaviour related to autism core symptoms is—through neuroanatomical variation—associated with key aetiological (genetic) mechanisms in autism. Moreover, we found that both cross-sectional and longitudinal outcome-related neuroanatomical variation was associated with genes linked to specific (developmental) neurobiological processes implicated in autism. For example, group differences in cortical thickness were enriched for genes preferentially expressed during prenatal periods in migrating excitatory cells, maturing excitatory cells enriched in upper layers, excitatory deep layer II cells [37]; GABAergic pathways [64]; and differences in surface area were enriched for microglial-expressed genes involved in immune functions [38]. However, we observed these enrichments only in adaptive Increasers and No-changers, and not in Decreasers. This is in line with results from previous studies in autistic toddlers, which examined early development in language ability (which may be linked to adaptive behaviour) [65, 66]. Specifically, these studies reported that better outcome was linked to variation in cortical thickness genetically enriched for prenatal excitatory cell types; and to variation in surface area genetically enriched for prenatal glial (including microglial) cells [65, 66]. Combined, our and these previous results suggest that the observed enrichments may indicate normative/compensatory mechanisms that help prevent or ‘rescue’ regression in adaptive behaviour.

Given that we compared neurotypicals to three (adaptive behaviour-based) autistic subgroups, we may have expected to consistently observe autism-related differences, possibly overshadowing/camouflaging any subgroups-specific atypicalities. Instead, we observed no overlap in the between-group differences, i.e. each autistic subgroup had its own (atypical) neurobiological profile. These results highlight the significant cross-sectional and longitudinal neurobiological and associated clinical (adaptive) heterogeneity, both between neurotypical and autistic individuals as a whole group and within the autism spectrum. This has implications for future clinical trials; especially given that adaptive behaviour has been recommended (by researchers and stakeholders [8])—and is increasingly used [67, 68]—as an endpoint in intervention studies. For example, our results suggest that future clinical trials which use adaptive outcome as an endpoint should consider stratifying their participants into neurobiologically and or clinically homogeneous subgroups. By using our results (once they are validated), these studies could parse autism heterogeneity to identify groups of interest (e.g. those individuals less likely to improve regardless of interventions) and thereby advance ‘precision medicine’.

Notably, the specificity of our results (i.e. the identified regions and associated genes) to adaptive (vs other cognitive-behavioural) outcomes remains to be explored. Specifically, we observed neuroanatomical differences in large brain regions, many of which have been linked not only to adaptive behaviour and autism, but also to other cognitive functions. This included differences in the anterior cingulate cortex, which has also been implicated in repetitive behaviour [69], a core symptom of autism. Similarly, we observed differences in the cuneus and the lateral occipital cortex, which have been linked to sensory (e.g. visual) processing [70]. A potential explanation for this observation is that adaptive outcome is underpinned by networks of brain regions that subserve not only social-communication processing but also other (autism-related) features. This is in line with the fact that, although adaptive behaviour has been strongly associated with social-communication, it is a composite measure that also incorporates aspects such as motor function, sensory processing, restricted and repetitive behaviours, and symptoms of psychiatric conditions (e.g. inattention and hyperactivity in attention-deficit/hyperactivity disorder [ADHD]) [71]. Alternatively, our findings may reflect that, during the observed time period, autistic individuals changed not only in adaptive behaviour but also in other (related) cognitive-behavioural features; and each of these outcomes may also be associated with a neuroanatomical profile. This is in line with our secondary findings that neuroanatomical differences between the ‘original’ subgroups overlapped spatially with differences between subgroups derived using alternative clinical and behavioural features, e.g. restricted/repetitive behaviours. Nonetheless, additional research is required to determine the specificity of our observed neuroanatomical differences to variation in adaptive outcome. Similarly, it is unclear if the genomic factors associated with these neuroanatomical differences are specific to adaptive outcome-related neuroanatomy. For instance, we identified enrichment for genes related to migrating and maturing excitatory cells and to GABAergic pathways. However, previous studies have shown that excitatory pyramidal cells represent the majority (~75–89%) of neurons in the cortex [72] and may therefore be implicated in autism regardless of the specific clinical outcome. Similarly, altered excitation-inhibition (e.g. glutamatergic-GABAergic) systems are thought to be a central element in the neurobiology of autism [20, 73–76]; and may therefore also underpin a broad range of functions other than adaptive behaviour. In fact, this prior work, together with the known interaction between different behavioural domains/cognitive functions (and the spatial overlap in the associated neuroanatomical profiles we detected), suggest that it is unlikely that genetically determined mechanisms underpinning differences in neurodevelopment are specific to adaptive outcome in autism.

Our results need to be considered in view of several methodological considerations and limitations that need to be addressed before our results can be applied in the clinic. Principal among these is age. Our sample included individuals ranging from childhood to adulthood. Selecting such a broad age-range was a conscious decision made for the following reason: unlike previous (longitudinal) studies of neuroanatomy (and associated genetic variation) that were restricted to individual age groups (e.g. [63]), including individuals from childhood to adulthood provided us with the unique opportunity to capture the relationship between neuroanatomical and clinical autism phenotypes *across different* developmental stages. Also, using a dimensional approach to study the impact of age helped us avoid potential pitfalls of a categorical approach. For instance, the latter relies on (arbitrary) age-cutoffs at the group-level, which may not relate to the developmental status of individuals. Nonetheless, we acknowledge that, given the developmental nature of autism, the relationship between adaptive outcome and neuroanatomy may be age-dependent; for instance, it is possible (and perhaps expected) that a developmental period of 1–2 years may hold a different significance in a 6-year-old compared to a 30-year-old person. To account for this, we rigorously corrected our analyses for (linear and quadratic) age, follow-up duration, and their interaction. Also, to examine the age-dependency of our discovered effects further, we stratified our sample by age groups (children, adolescents, and adults). However, these results should be interpreted with caution: this is because our stratification yielded unbalanced samples. Hence, it is unclear if these results reflect real biological developmental differences (i.e. the fact that between-group differences are differently prominent in younger/older participants); or if they stem from differences in sample sizes and resulting differences in variance.

Second, the investigated follow-up duration was limited to 12–24 months. This opportunity to examine neuroanatomical and clinical development in autism longitudinally (i.e. using repeated-measures within the same individuals) was unprecedented, given the scarcity of other comparable datasets and the challenges inherent to collecting large-scale longitudinal samples (e.g. cost, logistics, participant drop-out etc.). Nonetheless, in view of the developmental nature of autism, longer follow-up periods would be desirable to further trace developmental trajectories in this condition. To address this limitation, we are currently collecting additional follow-up data from a third timepoint.

Further steps that will move us towards being able to apply our results in the clinic include a replication of our results in an independent sample. The main reason for why we have not yet been able to do this is the specific design of our study (longitudinal collection of multimodal data) and our sample (a heterogeneous group of neurotypical and autistic individuals [men and women] across age, cognitive abilities [e.g. including intellectual disability], and with a range of co-occurring conditions). Specifically, while the study design and sample represent a strength of our project (as they enabled us to answer a novel question in a uniquely suited dataset), they also prevented us from identifying a comparable dataset to attempt a replication of our findings. We aim to do this once suitable datasets become available.

Taken together, these future steps will help consolidate our results in different subgroups along the autism spectrum and thereby establish the context of use in which our results may be applicable (e.g. in children/adults) in the clinic. Combined, such studies will provide a basis for the future development of clinical interventions that target the mechanisms associated with specific (e.g. relatively poor adaptive) clinical outcomes.

### Supplementary information

Supplementary Material

## Data Availability

To examine genetic enrichment (as described in the Methods), we used a script that is available at github.com/mvlombardo/utils/blob/master/genelistOverlap.R.
