# Supplementary material for: Cross-sectional and longitudinal neuroanatomical profiles of distinct clinical (adaptive) outcomes in autism
Source: Mol Psychiatry. 2023 Mar 29;28(5):2158–69. doi: 10.1038/s41380-023-02016-z (PMC10575772; doi:10.1038/s41380-023-02016-z)
Supplement: Supplementary file 1 — Supplementary Material [file 41380_2023_2016_MOESM1_ESM.docx]

**Supplement**

**Materials and Methods**

*Participants and procedure*

Our data was part of the Longitudinal European Autism Project (LEAP) described in (1). We included participants if they or their parents/guardians were able to provide informed written or verbal consent/assent to their participation in this study. Autistic participants were included if they had an existing autism diagnosis in line with DSM-IV/ICD-10 or DSM-5 criteria. As most autistic individuals have one or more co-occurring psychiatric conditions, all psychiatric comorbidities (except for psychosis and bipolar disorders) were allowed (1). We also included individuals with intellectual disability in both groups. Further, given the high number of autistic individuals with prescriptions for regular medication (30-50% in Europe and 70% in the USA), we included participants on stable medication (1). In contrast, we excluded participants with conditions preventing safe scanning (e.g., metal objects in their body). Our study was approved by national and local ethics review boards at all study sites. These included the London-Central and Queen Square Health Research Authority Research Ethics Committee (University of Cambridge and King’s College London; ID 13/LO/1156), the UMM University Medical Mannheim Medical Ethics Commission II (Mannheim University; ID 2014-540N-MA), the Radboud University Medical Centre Institute Ensuring Quality and Safety Committee on Research Involving Human Subjects Arnhem-Nijmegen (Radboud University and Utrecht University; ID 2013/455), and the University Campus Bio-Medical Ethics Committee De Roma (Rome University; ID 18/14 PAR ComET CBM). This study was carried out to Good Clinical Practice (ICH GCP) standards.

*Measures of adaptive functioning using the VABS-II*

To assess the autistic participants’ adaptive behaviour, we interviewed their parents/carers using the VABS-II (2). As previously described (3), this semi-structured interview (test-retest reliability: 0.8-0.9) assesses a person’s current level of everyday functioning across three domains, each of which contains three subdomains. The domains include communication (subdomains: expressive, receptive, and written), daily living skills (community, domestic, and personal), and socialization (coping skills, interpersonal relationships, and play and leisure time). We calculated age-normed standard scores (mean=100, standard deviation=15) for each domain and generated composite scores (i.e., total degree of impairment across all three domains) at T1 and T2. We then quantified the change between T1 and T2 (∆=T2-T1) and used recently published estimates of what constitutes a ‘Minimal Clinically Important Difference’ (MCID) (4), to group autistic individuals into three adaptive clinical outcome groups: those whose scores could be said to meaningfully improve (“Increasers”; ∆V≥4), showed no meaningful change/stasis (“No-changers”; -4<∆V<4), and those whose scores declined (“Decreasers”; -4≥∆V). Unlike other, related, measures (e.g., the Minimum Detectable Change [MDC], which is based on the standard error of measurement), the MCID quantifies the amount of change required to be clinically, rather than statistically, meaningful. Accordingly, the MCID has been supported as a means to evaluate (treatment) outcomes, including by the Food and Drug Administration (FDA) (5).

*Cortical reconstruction using FreeSurfer*

Images were (pre)processed using the automated longitudinal stream in FreeSurfer v6.0 ([surfer.nmr.mgh.harvard.edu](https://surfer.nmr.mgh.harvard.edu/)). FreeSurfer uses robust, inverse consistent registration to create an unbiased within-subject template space (base image). Common information from this base image is then used to perform pre-processing (e.g., skull stripping, transformation to Talairach space etc.), which significantly increases reliability and statistical power (compared to using information from a single timepoint only) (6). Using the pre-processed data, we reconstructed cortical surfaces through well-validated and fully automated procedures (described in detail elsewhere, e.g., (7, 8)). Surfaces were inspected visually for reconstruction errors by three independent, experienced researchers (blinded to participants’ diagnostic status). Their ratings were as follows: at baseline, of an initial 709 scans, 347 (49%) were accepted, 52 (7%) rejected, mostly due to severe (motion) artefacts; and 310 (44%) were prescribed manual edits. Of these 310, 307 images improved after manual editing and reprocessing; while the remaining three did not improve and were therefore excluded from further analyses. A further 15 scans were excluded due to scanner upgrades and missing demographic information. In sum, we retained 639 scans. At follow-up, we obtained 459 scans. Of these, 286 (62%) were accepted, 22 (5%) rejected, and 151 (33%) prescribed manual edits. Of these 151, 142 images improved and were included; and nine did not improve and were excluded. Hence, at follow-up, we retained 428 images. Next, we excluded all participants who did not have both T1 and T2 structural data; and those autistic individuals who did not have both T1 and T2 adaptive behavioural data. Our final sample consisted of 333 individuals (161 autistic and 172 neurotypical individuals).

Next, we computed vertex-wise measures and whole-brain averages (used as covariates in subsequent statistical analyses) of surface area (as outlined by Winkler et al. (9)) and cortical thickness (calculated as the closest distance from the grey-white matter boundary to the grey matter-cerebrospinal fluid boundary at each vertex on the tessellated surface (8)). Additionally, we quantified neuroanatomical development between T1 and T2 using FreeSurfer’s “symmetrized percentage change” (SPC). The SPC captures the vertex-wise rate of change in feature X with respect to the average of feature X across time (T) points (T1 and T2)), i.e., $SPC=(100\times\left[ \left( X_{T2}- X_{T2} \right)\div\left( T_{T2}-T_{T1} \right) \right])\div\left[ 0.5*\left( X_{T1}+ X_{T2} \right) \right]$. Previous studies suggest that the SPC is more robust and has greater statistical power when examining change between time points than commonly used alternative measures, such as the difference (T2-T1), ratio (T2/T1), or percent change (100*(T2-T1/T1)) (10). However, the SPC (compared to e.g., the difference) hampers the use of standard statistical repeated-measures analyses. Hence, rather than employing a mixed-effects approach, we examined cross-sectional and longitudinal neuroanatomy separately.

All features were smoothed (10 mm surface-based kernel) according to previously published recommendations (11). Given that our data was collected across multiple study centres, we also applied ComBat batch effect harmonization (12) to account for site effects prior to statistical modelling.

*Neuroimaging analyses*

*Primary analyses*

First, we examined differences in neuroanatomy at T1 (baseline) between the neurotypicals and each outcome group. To this aim, we ran separate general linear regression models (GLM) computing the parameter estimates for surface area (SA_i_) or cortical thickness (CT_i_) at each vertex *i*. We included group, and sex as fixed-effect factors; and linear (surface area/cortical thickness) and quadratic (cortical thickness) age at T1 (as in e.g., (3)), IQ, and total brain measures (total surface area, mean cortical thickness), as continuous covariates, i.e.,

$\mathrm{SA}_{i}=\beta_{0}+\beta_{1}AgeT1+\beta_{2}Group+\beta_{3}Sex+\beta_{4}IQ+\beta_{5}Total Brain+\varepsilon_{i}$ and

$\mathrm{CT}_{i}=\beta_{0}+\beta_{1}AgeT1+\beta_{2}{AgeT1}^{2}+\beta_{3}Group+\beta_{4}Sex+\beta_{5}IQ+\beta_{6}Total Brain+\varepsilon_{i}$, where $\varepsilon_{i}$ is the residual error at vertex *i*. We examined between-group differences using the coefficients β_2_ (for surface area) and β_3_ (for cortical thickness).

Second, we examined differences in intra-individual change in neuroanatomy between T1 and T2 between the neurotypicals and each outcome group. We used separate GLMs that included the model terms above and also corrected for the interaction between age at T1 and the follow-up duration (∆T). Note that we did not include ∆T and ‘baseline neuroanatomy’ in our models because these factors are already accounted for in the initial computation of the SPC. Hence, $\Delta Feature=\beta_{0}+\beta_{1}AgeT1+\beta_{2}AgeT1*\Delta T+\beta_{3}Group+\beta_{4}Sex+\beta_{5}IQ+\beta_{7}Total Brain {+ \varepsilon}_{i}$. We evaluated between-group differences using the coefficient β_3_.

Third, we investigated differences in neuroanatomy at T2 (follow-up) between the neurotypicals and each outcome group. We performed separate GLMs as specified above, while correcting for age at T2, i.e., $\mathrm{SA}_{i}= \beta_{0}+\beta_{1}AgeT2+\beta_{2}Group+\beta_{3}Sex+\beta_{4}IQ+\beta_{5}Total Brain+\varepsilon_{i}$ and $\mathrm{CT}_{i}=\beta_{0}+\beta_{1}AgeT2+\beta_{2}{AgeT2}^{2}+\beta_{3}Group+\beta_{4}Sex+\beta_{5}IQ+\beta_{6}Total Brain+\varepsilon_{i}$.

Continuous variables were mean centred across groups to improve the interpretability of the GLM coefficients. We corrected for multiple comparisons across the whole brain using random-field theory (RFT)-based cluster-correction for non-isotropic images (cluster-forming and cluster-p value threshold both <.01, two-tailed) (13). Surface area and cortical thickness are thought to have distinct neurobiological underpinning mechanisms (e.g., (14)), hence we treated them as separate analyses and did not correct for multiple comparisons across these two features. Further, we did not correct for multiple comparisons across the three subgroups, as we treated them as clinically separate. To visualize our results, we mapped our statistical effects onto the FreeSurfer high-resolution template in standard space (fsaverage).

*Secondary analyses*

To establish the robustness of our results in view of additional potential confounders, we repeated our analyses i) while correcting for medication (yes vs unknown/no medication), ii) while not controlling for total brain measures, and iii) while excluding individuals with intellectual disability. Also, to explore the generalizability of our results to other cognitive-behavioural features associated with adaptive behaviour, we repeated our analyses using different approaches to stratify autistic individuals into clinical subgroups. In particular, we grouped individuals into “Increasers”, “No-changers” and “Decreasers” based on change in i) each of the VABS-II domains, i.e., communication, daily living, and social skills; ii) the ADOS social domain; and iii) the ADOS restricted and repetitive behaviour domain. To group individuals based on VABS-II domain outcomes, we applied the same MCID-based cut-off (∆≥4 units) as in our primary analyses. Note that the MCID has not yet been validated for domain scores; hence, our findings should be considered preliminary. To group individuals based on ADOS domain outcomes, we computed change scores in each ADOS domain (∆ADOS=ADOS_T2_-ADOS_T1_), standardized change scores. To obtain balanced group numbers, we applied a cut-off of 0.33 standard deviations (SD) to identify Decreasers (∆ADOS≤-0.33SD), No-changers (-0.33SD <∆ADOS<0.33SD) and Increasers (∆ADOS ≤0.33SD). To evaluate the association between adaptive outcome and neuroanatomy using a dimensional (rather than categorical) approach, we assessed the effect of change in adaptive behaviour on neuroanatomy across autistic subgroups. Finally, to explore the impact of age on our between-group differences, we repeated our primary analyses within individual age-bands (children: 6-11 years; adolescents: 12-17 years; and adults: 18-30 years). Note that this yielded the following sample numbers per subgroup: TD: 48 children, 67 adolescents, 57 adults; IG: 7 children, 24 adolescents, 35 adults; NCG: 11 children, 25 adolescents, 6 adults; DG: 14 children, 16 adolescents, 23 adults). Because of this sample imbalance, we decided not to include these results in the main manuscript, but in the supplement; and we recommend caution in their interpretation.

*Genomic analyses (virtual histology)*

*Primary analyses*

Next, we aimed to link our neuroanatomical results to putative genomic (genetic and transcriptomic) mechanisms. As previously described in detail (3), we first identified genes expressed in spatial patterns similar to the neuroanatomical differences between autistic subgroups and neurotypicals (decoding). Second, we tested how these genes overlapped with genes that have previously been associated with autism at the genetic and transcriptomic level (15, 16, 17, 18) and that we have previously linked to cross-sectional neuroanatomical variation in autism (3) (enrichment). Decoding: To identify genes expressed throughout the brain in spatial patterns similar to our regions of interest (i.e., the neuroanatomical differences between autistic subgroups and neurotypicals), we leveraged Neurovault (<https://neurovault.org>), python code embedded within Neurovault and Neurosynth (<https://neurosynth.org>), and the Allen Human Brain Atlas (AHBA) (19). The AHBA is publicly available and currently the most comprehensive atlas of gene-expression in the brain. Nonetheless, it is limited in its sample (adult donors only) and spatial coverage/resolution. Hence, future studies should repeat our analyses using age-specific high-resolution gene-expression atlases once they become available. Enrichment: To examine the relevance and roles of the identified genes, we leveraged R code written by M. V. L. ([github.com/mvlombardo/utils/blob/master/genelistOverlap.R](https://github.com/mvlombardo/utils/blob/master/genelistOverlap.R)). Specifically, we tested for enrichment of genes associated with autism at the genetic level, including the 102 rare de novo protein truncating variants identified in the largest exome sequencing study of autism world-wide (15) (ASD dnPTVs); and an autism-related gene list compiled by SFARI (ASD SFARI; categories S, 1, 2, & 3 downloaded on January 14^th^, 2020 from [gene.sfari.org](https://gene.sfari.org/)). We also examined enrichment of genes associated with autism at the transcriptomic level. This included a list of differentially expressed (upregulated/downregulated) genes identified in autism post-mortem frontal and temporal cortex tissue (ASD DE Downreg, ASD DE Upreg) (16); and lists of differentially expressed genes identified in schizophrenia (SCZ DE) and bipolar disorder (BD DE), as these conditions are somewhat genetically correlated with autism (16). Also, this included genes differentially expressed in autism in specific cell types (ASD: Excitatory, Inhibitory, Microglia, Oligodendrocyte, Astrocyte, Endothelial) (17). Last, we examined genes from differentially expressed co-expression modules in autism (ASD CTX Downreg CoExpMods, ASD CTX Upreg CoExpMods) (18). We corrected our analyses for multiple comparisons across all subgroup contrasts and gene sets (p_FDR_<.05).

*Secondary analyses*

We based our primary analyses on a total background list of all 20,787 genes considered in Neurosynth (<https://neurosynth.org>)). However, this broad background may have biased our results towards identifying genes that are expressed in brain in general, rather than identifying genes associated with the observed neuroanatomical differences in particular. To address this limitation, we repeated our analyses while restricting the background list to those 16,906 genes specifically estimated to be expressed in cortical tissue (20).

Also, our primary analyses focused on identifying genes associated with autism in general. To extend our results, we conducted secondary analyses where we tested the association between the observed neuroanatomical differences and specific (developmentally relevant) cell-types and neurobiological processes linked to both autism and adaptive behaviour. Specifically, we examined enrichment for three gene sets of interest. The first set included genes expressed prenatally in specific cell types, including endothelial, excitatory deep layer I/II, maturing excitatory, maturing excitatory upper-layer enriched, migrating excitatory, interneuron CGE, interneuron medial ganglionic-eminence [MGE], intermediate progenitors, microglia, oligodendrocyte precursor, outer radial glia, pericyte, cycling progenitor G2M phase, cycling progenitor S phase, and ventricular radial glia cells (21). We chose to examine these genes that are thought to play preferential roles in prenatal (mid-gestational) periods (21), because many processes that occur during this developmental epoch are implicated in developmental conditions such as autism. The second set comprised genes associated with excitatory (glutamate/glutamine) and inhibitory (GABA) pathways (22). We chose these genes because excitation-inhibition systems are increasingly emerging as key pathological pathways in autism (23, 24, 25, 26, 27, 28, 29); and have been linked to behaviours and cognitive processes that influence adaptive behaviour (e.g., (30)). The third set contained immune genes expressed in microglia (31). We selected these genes because a growing body of literature implicates the immune system (including microglia, the brain’s primary innate immune effector cells) in the aetiology of autism (32). Specifically, we selected microglia-expressed genes reported to influence neurodevelopmental processes related to cognition by modulating neuroanatomy (31).

**Supplementary Results**

*Neuroanatomical differences*

*Increasers > Neurotypicals*

Increasers showed no significant cross-sectional or longitudinal differences in SA compared to the neurotypicals. Increasers (vs neurotypicals) had lower CT at T1 in a cluster in the right precentral gyrus (BA4; T=-2.34). Between T1 and T2, Increasers (vs neurotypicals) displayed no differences in CT development. Last, Increasers (vs neurotypicals) showed lower CT at T2 in a cluster in the right premotor cortex and supplementary motor area (BA6; T=-2.34) (Fig. 1, table S1).

*No-changers vs Neurotypicals*

No-changers (vs neurotypicals) displayed greater SA at T1 in a cluster in the right temporal pole (BA38; T=3.23). Between T1 and T2, No-changers (vs neurotypicals) displayed an accelerated growth of SA in clusters in the right precentral gyrus (BA6; T=4.40) and right postcentral gyrus (BA1; T=5.35). Also, No-changers on average had a growth (while neurotypicals had a shrinking) of SA in a cluster in the right inferior parietal cortex (BA39; T=4.11). Last, No-changers (vs neurotypicals) showed an accelerated shrinking in SA in a cluster in the left caudal middle frontal gyrus and pars opercularis (BA44; T=-2.35). No-changers showed no differences in SA at T2 (Fig. 2, table S2).

Also, No-changers (vs neurotypicals) showed no differences in CT at T1. Between T1 and T2, No-changers showed a growth (while neurotypicals displayed a decline) in CT in clusters in the left superior frontal gyrus (BA6; T=4.48) and left posterior cingulate cortex (BA24; T=3.45). In contrast, No-changers displayed a shrinking (while neurotypicals showed a growth) in CT in clusters in the right superior parietal cortex and cuneus cortex (BA19; T=-2.35), right superior parietal cortex (BA39/40; T=-2.35), left superior parietal cortex (BA19; T=-2.35), and right inferior parietal cortex (BA39; T=-2.35). Also, No-changers (vs neurotypicals) displayed an accelerated decrease in CT in a cluster in the left lateral occipital cortex (BA37; T=-2.34). At T2, we observed no CT differences in the Increasers vs the neurotypicals (Fig. 2, table S2).

*Decreasers vs Neurotypicals*

Decreasers (vs neurotypicals) displayed greater SA at T1 in a cluster in the right superior temporal gyrus (BA38; T=3.80); and lower SA at T1 in a cluster in the right caudal anterior-cingulate cortex (BA24; T=-2.34). Between T1 and T2, Decreasers (vs neurotypicals) displayed an accelerated reduction in SA in a cluster in the left superior parietal cortex (BA39; T=-2.34). Also, Decreasers displayed a shrinking (while neurotypicals showed a growth) of SA in clusters in the left lateral occipital cortex (BA18; T=-2.35) and left parahippocampal gyrus (BA37; T=-2.35) (Fig. 3, table S3).

Decreasers (vs neurotypicals) had greater CT at T1 in a cluster in the left dorsal anterior cingulate cortex (BA32; T=3.82); and lower CT at T1 in a cluster in the left parahippocampal gyrus (BA36; T=-2.34). Between T1 and T2, Decreasers (compared to neurotypicals) displayed no differences in CT development. Further, Decreasers (vs neurotypicals) had lower CT at T2 in a cluster in the right precentral gyrus (BA6; T=-2.34) (Fig. 3, table S3).

Table S1 Regions displaying significant neuroanatomical differences between neurotypicals and those individuals whose adaptive behavioural scores increased. Abbreviations: BA, Brodmann area; H, hemisphere; L, left; ns, not significant; R, right; T_max/min_, maximal/minimal T-value. Coordinates are presented in Talairach space.

| ID | H | Region | T_max/min_  [x, y, z] | | | T max | Vertices | Cluster probability | BA |
| --- | --- | --- | --- | --- | --- | --- | --- | --- | --- |
| Increasers > Neurotypicals | | | | | | | | | |
| Surface area | | | | | | | | | |
| T1 | | | | | | | | | |
| 1 |  | ns |  |  |  |  |  |  |  |
| ∆T | | | | | | | | | |
| 1 |  | ns |  |  |  |  |  |  |  |
| T2 | | | | | | | | | |
| 1 |  | ns |  |  |  |  |  |  |  |
| Cortical thickness | | | | | | | | | |
| T1 | | | | | | | | | |
| 1 | R | Precentral gyrus | 36 | -12 | 34 | -2.34 | 1273 | 1.28 e-03 | 4 |
| ∆T | | | | | | | | | |
| 1 |  | ns |  |  |  |  |  |  |  |
| T2 | | | | | | | | | |
| 1 | R | Premotor cortex and supplementary motor area | 44 | 4 | 39 | -2.34 | 1818 | 2.37 e-05 | 6 |

Table S2 Regions displaying significant neuroanatomical differences between neurotypicals and those individuals whose adaptive behavioural scores did not change. Abbreviations: BA, Brodmann area; H, hemisphere; L, left; ns, not significant; R, right; T_max/min_, maximal/minimal T-value. Coordinates are presented in Talairach space.

| ID | H | Region | T_max/min_  [x, y, z] | | | T max | Vertices | Cluster probability | BA |
| --- | --- | --- | --- | --- | --- | --- | --- | --- | --- |
| No-changers> Neurotypicals | | | | | | | | | |
| Surface area | | | | | | | | | |
| T1 | | | | | | | | | |
| 1 | R | Temporal pole | 31 | 10 | -31 | 3.23 | 533 | 4.43 e-03 | 38 |
| ∆T | | | | | | | | | |
| 1 | R | Precentral gyrus | 35 | -12 | 60 | 4.40 | 1078 | 4.39 e-04 | 6 |
| 2 | R | Postcentral gyrus | 32 | -31 | 55 | 5.35 | 624 | 1.48 e-03 | 1 |
| 3 | R | Inferior parietal cortex | 48 | -52 | 43 | 4.11 | 497 | 3.27 e-03 | 39 |
| 4 | L | Caudal middle frontal gyrus  Pars opercularis | -39 | 7 | 19 | -2.35 | 983 | 7.08 e-04 | 44 |
| T2 | | | | | | | | | |
| 1 |  | ns |  |  |  |  |  |  |  |
| Cortical thickness | | | | | | | | | |
| T1 | | | | | | | | | |
| 1 |  | ns |  |  |  |  |  |  |  |
| ∆T | | | | | | | | | |
| 1 | L | Superior frontal gyrus | -11 | 14 | 42 | 4.48 | 1177 | 1.28 e-04 | 6 |
| 2 | L | Posterior cingulate cortex | -5 | -9 | 31 | 3.45 | 841 | 1.99 e-03 | 24 |
| 3 | R | Superior parietal cortex  cuneus cortex | 17 | -83 | 28 | -2.35 | 1076 | 5.73 e-06 | 19 |
| 4 | R | Superior parietal cortex | 36 | -36 | 35 | -2.35 | 1236 | 8.12 e-06 | 39/40 |
| 5 | L | Superior parietal cortex | -10 | -83 | 31 | -2.35 | 1171 | 2.40 e-05 | 19 |
| 6 | R | Inferior parietal cortex | 34 | -61 | 41 | -2.35 | 1104 | 2.17 e-04 | 39 |
| 7 | L | Lateral occipital cortex | -42 | -57 | -2 | -2.34 | 833 | 3.44 e-04 | 37 |
| T2 | | | | | | | | | |
| 1 |  | ns |  |  |  |  |  |  |  |

Table S3 Regions displaying significant neuroanatomical differences between neurotypicals and those individuals whose adaptive behavioural scores decreased. Abbreviations: BA, Brodmann area; H, hemisphere; L, left; ns, not significant; R, right; T_max/min_, maximal/minimal T-value. Coordinates are presented in Talairach space.

| ID | H | Region | T_max/min_  [x, y, z] | | | T max | Vertices | Cluster probability | BA |
| --- | --- | --- | --- | --- | --- | --- | --- | --- | --- |
| Decreasers > Neurotypicals | | | | | | | | | |
| Surface area | | | | | | | | | |
| T1 | | | | | | | | | |
| 1 | R | Superior temporal gyrus | 44 | 8 | -21 | 3.80 | 954 | 6.19 e-04 | 38 |
| 2 | R | Caudal anterior-cingulate cortex | 5 | 16 | 21 | -2.34 | 376 | 6.49 e-03 | 24 |
| ∆T | | | | | | | | | |
| 1 | L | Superior parietal cortex | -31 | -65 | 33 | -2.34 | 768 | 1.80 e-03 | 39 |
| 2 | L | Lateral occipital cortex | -20 | -97 | 2 | -2.35 | 467 | 4.46 e-03 | 18 |
| 3 | L | Parahippocampal gyrus | -28 | -33 | -13 | -2.35 | 493 | 4.73 e-03 | 37 |
| T2 | | | | | | | | | |
| 5 |  | ns |  |  |  |  |  |  |  |
| Cortical thickness | | | | | | | | | |
| T1 | | | | | | | | | |
| 1 | L | **Dorsal anterior cingulate cortex** | -6 | 30 | -6 | 3.82 | 305 | 1.59 e-04 | 32 |
| 2 | L | **Parahippocampal gyrus** | -20 | -10 | -26 | -2.34 | 398 | 8.90 e-06 | 36 |
| ∆T | | | | | | | | | |
| 1 |  | ns |  |  |  |  |  |  |  |
| T2 | | | | | | | | | |
| 1 | R | Precentral gyrus | 28 | -14 | 64 | -2.34 | 1455 | 3.11 e-03 | 6 |

Table S4 Medication information for all participants included in this study (recorded at T1)

| **Medication** | **Decreasers** | **No-changers** | **Increasers** | **Neurotypicals** |
| --- | --- | --- | --- | --- |
| Yes | 24 | 23 | 18 | 12 |
| No | 15 | 14 | 23 | 63 |
| Unknown | 14 | 5 | 25 | 97 |
| *Nr of participants taking* | | | | |
| >1 drug | 10 | 3 | 7 | 2 |
| >2 drugs | 2 | 0 | 1 | 1 |
| *Medication type* | | | | |
| Antiepileptics | 2 | 2 | 1 | 2 |
| Antidepressants  SSRI  TCA  TeCA | 8  1  2 | 2  0  0 | 4  0  0 | 3  0  0 |
| Antimigraine preparations | 1 | 1 | 1 | 0 |
| Antipsychotics  Aripiprazole  Clozapine  Pipamperone  Risperidone | 3  0  1  1 | 0  0  0  3 | 0  1  0  1 | 0  0  0  0 |
| Anxiolytics | 0 | 0 | 1 | 0 |
| Drugs used in addictive disorder | 0 | 0 | 0 | 1 |
| Hypnotics and sedatives:  Melatonin | 8 | 8 | 9 | 1 |
| Other analgesics and antipyretics | 1 | 1 | 1 | 2 |
| Psychostimulants & other drugs used to treat ADHD  Atomoxetine  Dexamfetamine  Methylphenidate Hydrochloride | 1  0  6 | 0  0  5 | 1  1  3 | 1  0  3 |

Participants may have taken up to 2 different types of medication across the listed categories during study participation. Data based on the ATC classification system, Level-1 ATC code “N”.

# Supplementary Figures

Figure S1 Neuroanatomical differences between neurotypicals and those individuals whose adaptive behavioural scores increased. Each row displays unthresholded t-values. Abbreviations: L, left; R, right.

Figure S2 Neuroanatomical differences between neurotypicals and those individuals whose adaptive behavioural scores did not change. Each row displays unthresholded t-values. Abbreviations: L, left; R, right.

Figure S3 Neuroanatomical differences between neurotypicals and those individuals whose adaptive behavioural scores decreased. Each row displays unthresholded t-values. Abbreviations: L, left; R, right.

Figure S4 Neuroanatomical differences between neurotypicals and those individuals whose adaptive behavioural scores increased. Each row displays effect sizes (Cohen’s d). Positive/negative values indicate a positive/negative effect in terms of the contrasts shown (Increasers > Neurotypicals). Abbreviations: L, left; R, right.

Figure S5 Neuroanatomical differences between neurotypicals and those individuals whose adaptive behavioural scores did not change. Each row displays effect sizes (Cohen’s d). Positive/negative values indicate a positive/negative effect in terms of the contrasts shown (No-changers > Neurotypicals). Abbreviations: L, left; R, right.

Figure S6 Neuroanatomical differences between neurotypicals and those individuals whose adaptive behavioural scores decreased. Each row displays effect sizes (Cohen’s d). Positive/negative values indicate a positive/negative effect in terms of the contrasts shown (Decreasers > Neurotypicals). Abbreviations: L, left; R, right.

Figure S7 Neuroanatomical differences between neurotypicals and those individuals whose adaptive behavioural scores increased. Top row: primary results (random field theory (RFT)-corrected t-values). Middle row: results are corrected for medication (unthresholded t-values). Bottom row: results are not corrected for total brain values (unthresholded t-values). Abbreviations: CT, cortical thickness; L, left; R, right; T1, time point 1.

Figure S8 Neuroanatomical differences between neurotypicals and those individuals whose adaptive behavioural scores did not change. Top row: primary results (random field theory (RFT)-corrected t-values). Middle row: results are corrected for medication (unthresholded t-values). Bottom row: results are not corrected for total brain values (unthresholded t-values). Abbreviations: CT, cortical thickness; L, left; R, right; SA, surface area; T1, time point 1; ∆T, change between T1 and T2.

Figure S9 Neuroanatomical differences between neurotypicals and those individuals whose adaptive behavioural scores decreased. Top row: primary results (random field theory (RFT)-corrected t-values). Middle row: results are corrected for medication (unthresholded t-values). Bottom row: results are not corrected for total brain values (unthresholded t-values). Abbreviations: CT, cortical thickness; L, left; R, right; SA, surface area; T1, time point 1; T2, time point 2. ∆T, change between T1 and T2.

Figure S10 Neuroanatomical differences between neurotypicals and those individuals whose adaptive behavioural scores increased (excluding individuals with intellectual disability from our sample) (unthresholded t-values). Abbreviations: ID, intellectual disability; L, left; R, right.

Figure S11 Neuroanatomical differences between neurotypicals and those individuals whose adaptive behavioural scores did not change (excluding individuals with intellectual disability from our sample) (unthresholded t-values). Abbreviations: ID, intellectual disability; L, left; R, right.

Figure S12 Neuroanatomical differences between neurotypicals and those individuals whose adaptive behavioural scores decreased (excluding individuals with intellectual disability from our sample) (unthresholded t-values). Abbreviations: ID, intellectual disability; L, left; R, right.

Figure S13 Neuroanatomical differences in cortical thickness (CT) at T1 between neurotypicals and those individuals whose scores increased in adaptive behaviour (a), the VABS-II domains of communication (b), daily living (c), and social functioning (d); and the Autism Diagnostic Observation Schedule-2 Calibrated Severity Score in the social (e) and restricted and repetitive behaviour (RRB) (f) domain. Panel (a) displays random field theory (RFT)-corrected t-values. All other panels display unthresholded t-values. Abbreviations: L, left; R, right.

Figure S14 Neuroanatomical differences in cortical thickness (CT) at T2 between neurotypicals and those individuals whose scores increased in adaptive behaviour (a), the VABS-II domains of communication (b), daily living (c), and social functioning (d); and the Autism Diagnostic Observation Schedule-2 Calibrated Severity Score in the social (e) and restricted and repetitive behaviour (RRB) (f) domain. Panel (a) displays random field theory (RFT)-corrected t-values. All other panels display unthresholded t-values. Abbreviations: L, left; R, right.

Figure S15 Neuroanatomical differences in surface area (SA) at ∆T between neurotypicals and those individuals whose scores did not change in adaptive behaviour (a), the VABS-II domains of communication (b), daily living (c), and social functioning (d); and the Autism Diagnostic Observation Schedule-2 Calibrated Severity Score in the social (e) and restricted and repetitive behaviour (RRB) (f) domain. Panel (a) displays random field theory (RFT)-corrected t-values. All other panels display unthresholded t-values. Abbreviations: L, left; R, right.

Figure S16 Neuroanatomical differences in cortical thickness (CT) at ∆T between neurotypicals and those individuals whose scores did not change in adaptive behaviour (a), the VABS-II domains of communication (b), daily living (c), and social functioning (d); and the Autism Diagnostic Observation Schedule-2 Calibrated Severity Score in the social (e) and restricted and repetitive behaviour (RRB) (f) domain. Panel (a) displays random field theory (RFT)-corrected t-values. All other panels display unthresholded t-values. Abbreviations: L, left; R, right.

Figure S17 Neuroanatomical differences in surface area (SA) at T1 between neurotypicals and those individuals whose scores did not change in adaptive behaviour (a), the VABS-II domains of communication (b), daily living (c), and social functioning (d); and the Autism Diagnostic Observation Schedule-2 Calibrated Severity Score in the social (e) and restricted and repetitive behaviour (RRB) (f) domain. Panel (a) displays random field theory (RFT)-corrected t-values. All other panels display unthresholded t-values. Abbreviations: L, left; R, right.

Figure S18 Neuroanatomical differences in surface area (SA) at ∆T between neurotypicals and those individuals whose scores decreased in adaptive behaviour (a), the VABS-II domains of communication (b), daily living (c), and social functioning (d); and the Autism Diagnostic Observation Schedule-2 Calibrated Severity Score in the social (e) and restricted and repetitive behaviour (RRB) (f) domain. Panel (a) displays random field theory (RFT)-corrected t-values. All other panels display unthresholded t-values. Abbreviations: L, left; R, right.

Figure S19 Neuroanatomical differences in surface area at T1 between neurotypicals and those individuals whose scores decreased in adaptive behaviour (a), the VABS-II domains of communication (b), daily living (c), and social functioning (d); and the Autism Diagnostic Observation Schedule-2 Calibrated Severity Score in the social (e) and restricted and repetitive behaviour (RRB) (f) domain. Panel (a) displays random field theory (RFT)-corrected t-values. All other panels display unthresholded t-values. Abbreviations: L, left; R, right.

Figure S20 Neuroanatomical differences in cortical thickness (CT) at T1 between neurotypicals and those individuals whose scores decreased in adaptive behaviour (a), the VABS-II domains of communication (b), daily living (c), and social functioning (d); and the Autism Diagnostic Observation Schedule-2 Calibrated Severity Score in the social (e) and restricted and repetitive behaviour (RRB) (f) domain. Panel (a) displays random field theory (RFT)-corrected t-values. All other panels display unthresholded t-values. Abbreviations: L, left; R, right.

Figure S21 Neuroanatomical differences in cortical thickness (CT) at T2 between neurotypicals and those individuals whose scores decreased in adaptive behaviour (a), the VABS-II domains of communication (b), daily living (c), and social functioning (d); and the Autism Diagnostic Observation Schedule-2 Calibrated Severity Score in the social (e) and restricted and repetitive behaviour (RRB) (f) domain. Panel (a) displays random field theory (RFT)-corrected t-values. All other panels display unthresholded t-values. Abbreviations: L, left; R, right.

Figure S22 Neuroanatomical regions associated with adaptive outcome across autistic subgroups (unthresholded t-values). Abbreviations: T1, time point 1; ∆T, change between T1 and T2; ∆VABS-II, change in adaptive behaviour measured using the vineland adaptive behaviour scale-2.

Figure S23 Neuroanatomical differences between neurotypicals and autistic outcome groups in surface area at timepoint 1, stratified by age-groups (unthresholded t-values). Abbreviations: SA, surface area; T1, timepoint 1.

Figure S24 Neuroanatomical differences between neurotypicals and autistic outcome groups in surface area development, stratified by age-groups (unthresholded t-values). Abbreviations: SA, surface area; ∆T, time between timepoint 1 and 2.

Figure S25 Neuroanatomical differences between neurotypicals and autistic outcome groups in surface area at timepoint 2, stratified by age-groups (unthresholded t-values). Abbreviations: SA, surface area; T2, timepoint 2.

Figure S26 Neuroanatomical differences between neurotypicals and autistic outcome groups in cortical thickness at timepoint 1, stratified by age-groups (unthresholded t-values). Abbreviations: CT, cortical thickness; T1, timepoint 1.

Figure S27 Neuroanatomical differences between neurotypicals and autistic outcome groups in cortical thickness development, stratified by age-groups (unthresholded t-values). Abbreviations: CT, cortical thickness; ∆T, time between timepoint 1 and 2.

Figure S28 Neuroanatomical differences between neurotypicals and autistic outcome groups in cortical thickness at timepoint 2, stratified by age-groups (unthresholded t-values). Abbreviations: CT, cortical thickness; T2, timepoint 2.

Figure S29 Genetic correlates of neuroanatomical variability analysed using a more restricted gene background list of 16,906 genes expressed in cortical tissue (vs 20,787 genes in the original analysis): Enrichment analyses for cortical phenotypes (y-axis, rows) by autism-associated gene lists (x-axis, columns). Tile colours indicate FDR q-values. Tile labels indicate enrichment odds ratios. Abbreviations: CT, cortical thickness; delta, change between T1 and T2; DG, Decreasers; IG, Increasers; NCG, No-changers; NT, Neurotypicals; SA, surface area; T1, time point 1; T2, time point 2.

Figure S30 Genetic correlates of neuroanatomical variability: Enrichment analyses for cortical phenotypes (y-axis, rows) by autism-associated gene lists (x-axis, columns) with a focus on genes predominantly expressed prenatally. Tile colours indicate FDR q-values. Tile labels indicate enrichment odds ratios. Abbreviations: CT, cortical thickness; delta, change between T1 and T2; DG, Decreasers; IG, Increasers; NCG, No-changers; NT, Neurotypicals; SA, surface area; T1, time point 1; T2, time point 2.

Figure S31 Genetic correlates of neuroanatomical variability: Enrichment analyses for cortical phenotypes (y-axis, rows) by autism-associated gene lists (x-axis, columns) with a focus on genes related to glutamate and GABA pathways. Tile colours indicate FDR q-values. Tile labels indicate enrichment odds ratios. Abbreviations: CT, cortical thickness; delta, change between T1 and T2; DG, Decreasers; IG, Increasers; NCG, No-changers; NT, Neurotypicals; SA, surface area; T1, time point 1; T2, time point 2.

Figure S32 Genetic correlates of neuroanatomical variability: Enrichment analyses for cortical phenotypes (y-axis, rows) by autism-associated gene lists (x-axis, columns) with a focus on microglial immune genes. Tile colours indicate FDR q-values. Tile labels indicate enrichment odds ratios. Abbreviations: CT, cortical thickness; delta, change between T1 and T2; DG, Decreasers; IG, Increasers; NCG, No-changers; NT, Neurotypicals; SA, surface area; T1, time point 1; T2, time point 2.

**EU-AIMS/AIMS-2-TRIALS consortium:**

Jumana Ahmad^1^, Sara Ambrosino^10^, Bonnie Auyeung^13^, Tobias Banaschewski^15^, Simon Baron-Cohen^13^, Sarah Baumeister^15^, Christian F. Beckmann^3^, Sven Bölte^16^, Thomas Bourgeron^11^, Carsten Bours^3^, Michael Brammer^1^, Daniel Brandeis^15^, Claudia Brogna^17^, Yvette de Bruijn^3^, Jan K. Buitelaar^3^, Bhismadev Chakrabarti^13^, Tony Charman^7^, Ineke Cornelissen^3^, Daisy Crawley^1^, Flavio Dell’Acqua^1^, Guillaume Dumas^12^, Sarah Durston^10^, Christine Ecker^4^, Jessica Faulkner^1^, Vincent Frouin^18^, Pilar Garces^19^, David Goyard^18^, Lindsay Ham^20^, Hannah Hayward^1^, Joerg Hipp^19^, Rosemary Holt^13^, Mark H. Johnson^9^, Emily J.H. Jones^9^, Prantik Kundu^21^, Meng-Chuan Lai^22^, Xavier Liogier D’Ardhuy^19^, Michael V. Lombardo^5^, Eva Loth^1^, David J. Lythgoe^23^, René Mandl^10^, Andre Marquand^3^, Luke Mason^1^, Maarten Mennes^3^, Andreas Meyer-Lindenberg^15^, Caroline Moessnang^15^, Nico Müller^15^, Declan G.M. Murphy^1^, Bethany Oakley^1^, Laurence O’Dwyer^3^, Marianne Oldehinkel^3^, Bob Oranje^10^, Gahan Pandina^24^, Antonio M. Persico^14^, Barbara Ruggeri^25^, Amber Ruigrok^13^, Jessica Sabet^1^, Roberto Sacco^17^, Antonia San José Cáceres^1^, Emily Simonoff^26^, Will Spooren^27^, Julian Tillmann^6^, Roberto Toro^11^, Heike Tost^15^, Jack Waldman^13^, Steve C.R. Williams^23^, Caroline Wooldridge^23^, and Marcel P. Zwiers^3^.

^16^Center for Neurodevelopmental Disorders, Karolinska Institutet; Stockholm, Sweden.

^17^Department of Paediatrics, The Catholic University of America, University Campus Bio-Medico; Rome, Italy.

^18^Neurospin Centre CEA; Gif sur Yvette, France.

^19^Roche Pharma Research and Early Development, Neuroscience, Ophthalmology and Rare Diseases, Roche Innovation Center Basel; Basel, Switzerland.

^20^Regulatory Affairs, Pharmaceutical Development, F. Hoffmann-La Roche Pharmaceuticals; Basel, Switzerland.

^21^Department of Radiology, Icahn School of Medicine at Mount Sinai; New York, New York, USA.

^22^Child and Youth Mental Health Collaborative, Centre for Addiction and Mental Health and The Hospital for Sick Children, Department of Psychiatry, University of Toronto; Toronto, Canada.

^23^Department of Neuroimaging, Institute of Psychiatry, Psychology and Neuroscience, King's College London; London, United Kingdom.

^24^Janssen Research & Development; Titusville, New Jersey, USA.

^25^Social, Genetic and Developmental Psychiatry Centre, Institute of Psychiatry, Psychology and Neuroscience, King's College London; London, United Kingdom.

^26^Department of Child and Adolescent Psychiatry, Institute of Psychology, Psychiatry and Neuroscience, King's College London; London, United Kingdom.

^27^Roche Pharmaceutical Research and Early Development, NORD Discovery and Translational Area, Roche Innovation Center Basel; Basel, Switzerland.

**Supplementary References**

1.	Loth E, Charman T, Mason L, Tillmann J, Jones EJH, Wooldridge C, et al. The EU-AIMS Longitudinal European Autism Project (LEAP): design and methodologies to identify and validate stratification biomarkers for autism spectrum disorders. Mol Autism. 2017;8:24.

2.	Sparrow SS, Balla DA, Cicchetti DV. Vineland II: Vineland adaptive behavior scales. American Guidance Service Publishing 2005.

3.	Pretzsch CM, Schafer T, Lombardo MV, Warrier V, Mann C, Bletsch A, et al. Neurobiological Correlates of Change in Adaptive Behavior in Autism. Am J Psychiatry. 2022:appiajp21070711.

4.	Chatham CH, Taylor KI, Charman T, Liogier D'ardhuy X, Eule E, Fedele A, et al. Adaptive behavior in autism: Minimal clinically important differences on the Vineland-II. Autism Res. 2018;11(2):270-83.

5.	Health USDo, Human Services FDACfDE, Research, Health USDo, Human Services FDACfBE, Research, et al. Guidance for industry: patient-reported outcome measures: use in medical product development to support labeling claims: draft guidance. Health Qual Life Outcomes. 2006;4:79.

6.	Reuter M, Schmansky NJ, Rosas HD, Fischl B. Within-subject template estimation for unbiased longitudinal image analysis. Neuroimage. 2012;61(4):1402-18.

7.	Dale AM, Fischl B, Sereno MI. Cortical surface-based analysis. I. Segmentation and surface reconstruction. Neuroimage. 1999;9(2):179-94.

8.	Fischl B, Sereno MI, Tootell RB, Dale AM. High-resolution intersubject averaging and a coordinate system for the cortical surface. Hum Brain Mapp. 1999;8(4):272-84.

9.	Winkler AM, Sabuncu MR, Yeo BTT, Fischl B, Greve DN, Kochunov P, et al. Measuring and comparing brain cortical surface area and other areal quantities. Neuroimage. 2012;61(4):1428-43.

10.	Berry DA, Ayers GD. Symmetrized percent change for treatment comparisons. The American Statistician. 2006;60(1):27-31.

11.	Liem F, Merillat S, Bezzola L, Hirsiger S, Philipp M, Madhyastha T, et al. Reliability and statistical power analysis of cortical and subcortical FreeSurfer metrics in a large sample of healthy elderly. Neuroimage. 2015;108:95-109.

12.	Johnson WE, Li C, Rabinovic A. Adjusting batch effects in microarray expression data using empirical Bayes methods. Biostatistics. 2007;8(1):118-27.

13.	Worsley KJ, Andermann M, Koulis T, MacDonald D, Evans AC. Detecting changes in nonisotropic images. Hum Brain Mapp. 1999;8(2-3):98-101.

14.	Rakic P. Specification of cerebral cortical areas. Science. 1988;241(4862):170-6.

15.	Satterstrom FK, Kosmicki JA, Wang J, Breen MS, De Rubeis S, An JY, et al. Large-Scale Exome Sequencing Study Implicates Both Developmental and Functional Changes in the Neurobiology of Autism. Cell. 2020;180(3):568-84 e23.

16.	Gandal MJ, Zhang P, Hadjimichael E, Walker RL, Chen C, Liu S, et al. Transcriptome-wide isoform-level dysregulation in ASD, schizophrenia, and bipolar disorder. Science. 2018;362(6420).

17.	Velmeshev D, Schirmer L, Jung D, Haeussler M, Perez Y, Mayer S, et al. Single-cell genomics identifies cell type-specific molecular changes in autism. Science. 2019;364(6441):685-9.

18.	Parikshak NN, Swarup V, Belgard TG, Irimia M, Ramaswami G, Gandal MJ, et al. Genome-wide changes in lncRNA, splicing, and regional gene expression patterns in autism. Nature. 2016;540(7633):423-7.

19.	Hawrylycz MJ, Lein ES, Guillozet-Bongaarts AL, Shen EH, Ng L, Miller JA, et al. An anatomically comprehensive atlas of the adult human brain transcriptome. Nature. 2012;489(7416):391-9.

20.	Richiardi J, Altmann A, Milazzo AC, Chang C, Chakravarty MM, Banaschewski T, et al. BRAIN NETWORKS. Correlated gene expression supports synchronous activity in brain networks. Science. 2015;348(6240):1241-4.

21.	Polioudakis D, de la Torre-Ubieta L, Langerman J, Elkins AG, Shi X, Stein JL, et al. A Single-Cell Transcriptomic Atlas of Human Neocortical Development during Mid-gestation. Neuron. 2019;103(5):785-801 e8.

22.	Naaijen J, Bralten J, Poelmans G, consortium I, Glennon JC, Franke B, et al. Glutamatergic and GABAergic gene sets in attention-deficit/hyperactivity disorder: association to overlapping traits in ADHD and autism. Transl Psychiatry. 2017;7(1):e999.

23.	Rubenstein JL, Merzenich MM. Model of autism: increased ratio of excitation/inhibition in key neural systems. Genes Brain Behav. 2003;2(5):255-67.

24.	Pretzsch CM, Freyberg J, Voinescu B, Lythgoe D, Horder J, Mendez MA, et al. Effects of cannabidiol on brain excitation and inhibition systems; a randomised placebo-controlled single dose trial during magnetic resonance spectroscopy in adults with and without autism spectrum disorder. Neuropsychopharmacology. 2019.

25.	Pretzsch CM, Voinescu B, Lythgoe D, Horder J, Mendez MA, Wichers R, et al. Effects of cannabidivarin (CBDV) on brain excitation and inhibition systems in adults with and without Autism Spectrum Disorder (ASD): a single dose trial during magnetic resonance spectroscopy. Transl Psychiatry. 2019;9(1):313.

26.	Pretzsch CM, Findon JL, Murphy DG. 17 Autism Spectrum Disorders in Adults. The Oxford Handbook of Adult Cognitive Disorders. 2019:359.

27.	Pretzsch CM, Floris DL. Balancing excitation and inhibition in the autistic brain. Elife. 2020;9.

28.	Pretzsch CM, Floris DL, Voinescu B, Elsahib M, Mendez MA, Wichers R, et al. Modulation of striatal functional connectivity differences in adults with and without autism spectrum disorder in a single-dose randomized trial of cannabidivarin. Mol Autism. 2021;12(1):49.

29.	Pretzsch CM, Voinescu B, Mendez MA, Wichers R, Ajram L, Ivin G, et al. The effect of cannabidiol (CBD) on low-frequency activity and functional connectivity in the brain of adults with and without autism spectrum disorder (ASD). J Psychopharmacol. 2019:269881119858306.

30.	Cochran DM, Sikoglu EM, Hodge SM, Edden RA, Foley A, Kennedy DN, et al. Relationship among Glutamine, gamma-Aminobutyric Acid, and Social Cognition in Autism Spectrum Disorders. J Child Adolesc Psychopharmacol. 2015;25(4):314-22.

31.	Corley E, Holleran L, Fahey L, Corvin A, Morris DW, Donohoe G. Microglial-expressed genetic risk variants, cognitive function and brain volume in patients with schizophrenia and healthy controls. Transl Psychiatry. 2021;11(1):490.

32.	Onore C, Careaga M, Ashwood P. The role of immune dysfunction in the pathophysiology of autism. Brain Behav Immun. 2012;26(3):383-92.
